# The association between Chinese visceral adiposity index and cardiometabolic multimorbidity among Chinese middle-aged and older adults: a national cohort study

**DOI:** 10.3389/fendo.2024.1381949

**Published:** 2024-03-27

**Authors:** Xiaomei Ye, Guangru Zhang, Chenyu Han, Ping Wang, Jiaping Lu, Min Zhang

**Affiliations:** ^1^ Department of Endocrinology, Qingpu Branch of Zhongshan Hospital Affiliated to Fudan University, Shanghai, China; ^2^ Department of General Practice, Community Health Service Center Xiayang Street, Shanghai, China

**Keywords:** Chinese visceral adiposity index, cardiometabolic multimorbidity, diabetes, heart disease, stroke, middle-aged and older adults, cohort study

## Abstract

**Objective:**

This study aimed to explore the association between the Chinese visceral adiposity index (CVAI) and cardiometabolic multimorbidity in middle-aged and older Chinese adults.

**Methods:**

The data used in this study were obtained from a national cohort, the China Health and Retirement Longitudinal Study (CHARLS, 2011-2018 wave). The CVAI was measured using previously validated biomarker estimation formulas, which included sex, age, body mass index, waist circumference, triglycerides, and high-density lipoprotein cholesterol. The presence of two or more of these cardiometabolic diseases (diabetes, heart disease, and stroke) is considered as cardiometabolic multimorbidity. We used Cox proportional hazard regression models to examine the association between CVAI and cardiometabolic multimorbidity, adjusting for a set of covariates. Hazard ratios (HRs) and 95% confidence intervals (CIs) were used to show the strength of the associations. We also conducted a subgroup analysis between age and sex, as well as two sensitivity analyses. Receiver operator characteristic curves (ROC) were used to test the predictive capabilities and cutoff value of the CVAI for cardiometabolic multimorbidity.

**Results:**

A total of 9028 participants were included in the final analysis, with a mean age of 59.3 years (standard deviation: 9.3) and women accounting for 53.7% of the sample population. In the fully-adjusted model, compared with participants in the Q1 of CVAI, the Q3 (HR = 2.203, 95% CI = 1.039 – 3.774) and Q4 of CVAI (HR = 3.547, 95% CI = 2.100 – 5.992) were associated with an increased risk of cardiometabolic multimorbidity. There was no evidence of an interaction between the CVAI quartiles and sex or age in association with cardiometabolic multimorbidity (*P >*0.05). The results of both sensitivity analyses suggested that the association between CVAI and cardiometabolic multimorbidity was robust. In addition, the area under ROC and ideal cutoff value for CVAI prediction of cardiometabolic multimorbidity were 0.685 (95% CI = 0.649-0.722) and 121.388.

**Conclusion:**

The CVAI is a valid biomarker with good predictive capability for cardiometabolic multimorbidity and can be used by primary healthcare organizations in the future for early warning, prevention, and intervention with regard to cardiometabolic multimorbidity.

## 1 Introduction

The prevalence of cardiometabolic diseases, such as cardiovascular diseases, diabetes, and stroke, is an important threat to human health (1), especially among middle-aged and older populations. In recent years, the rate of cardiometabolic multimorbidity (defined herein as the presence of two or more the following cardiometabolic diseases: diabetes, heart disease, and stroke) has increased due to rising global obesity rates (2, 3). A previous large survey of nearly 40,000 people in sub-Saharan Africa showed that the prevalence of cardiometabolic multimorbidity among respondents aged 15-69 years was 4.8% (4), with a prevalence of 14.1% in the 55-69 age group (4). In China, results from a population-based survey of 500,000 people aged 30-79 years showed that the prevalence of cardiometabolic multimorbidity was 6.0% (5). Moreover, multiple studies have confirmed that cardiometabolic multimorbidity is associated with the risk of cognitive decline and dementia (6), higher mortality (7, 8), higher depressive symptoms (9), and lower quality of life (10) compared to single cardiometabolic diseases. Given these health hazards and high prevalence trends, it is important to elucidate the potential risk factors for cardiometabolic multimorbidity and target these factors for early prevention and intervention, which may help reduce the incidence of cardiometabolic multimorbidity.

Evidences from basic biological research and observational studies have identified poor socioeconomic factors (e.g., low income, low education, and a single civil status), unhealthy lifestyles (e.g., smoking, alcohol consumption, and unhealthy diet), and gut metabolite as important factors related to cardiometabolic multimorbidity (11–16). Additionally, studies have explored the association between body measures, such as grip strength (17). Obesity is now widely recognized as the most relevant risk factor for cardiometabolic multimorbidity. A review of 16 cohort studies involving 0.12 million adults in Europe and the United States found that elevated BMI was associated with an increased risk of cardiometabolic multimorbidity (18). Obesity was an important risk factor for cardiometabolic multimorbidity in middle-aged and older people (19). Furthermore, visceral adipose tissue distribution, but not overall obesity, is an independent predictor of cardiometabolic disease (20). Previous Chinese scholars constructed a Chinese visceral adiposity index (CVAI) based on a sample of Chinese people using age, BMI, waist circumference, total triglycerides (TG), and high-density lipoprotein cholesterol (HDL-C) (21). The CVAI is a superior predictor of cardiometabolic diseases, such as stroke, diabetes, and cardiovascular disease, compared with BMI (22–24). However, it is unclear whether the CVAI predicts the development of cardiometabolic multimorbidity.

To fill the aforementioned gap in the literature and provide new biomarkers for the prevention of cardiometabolic multimorbidity, our study aimed to explore the association between CVAI and cardiometabolic multimorbidity based on a national cohort.

## 2 Materials and methods

### 2.1 Participants

**Figure 1:**
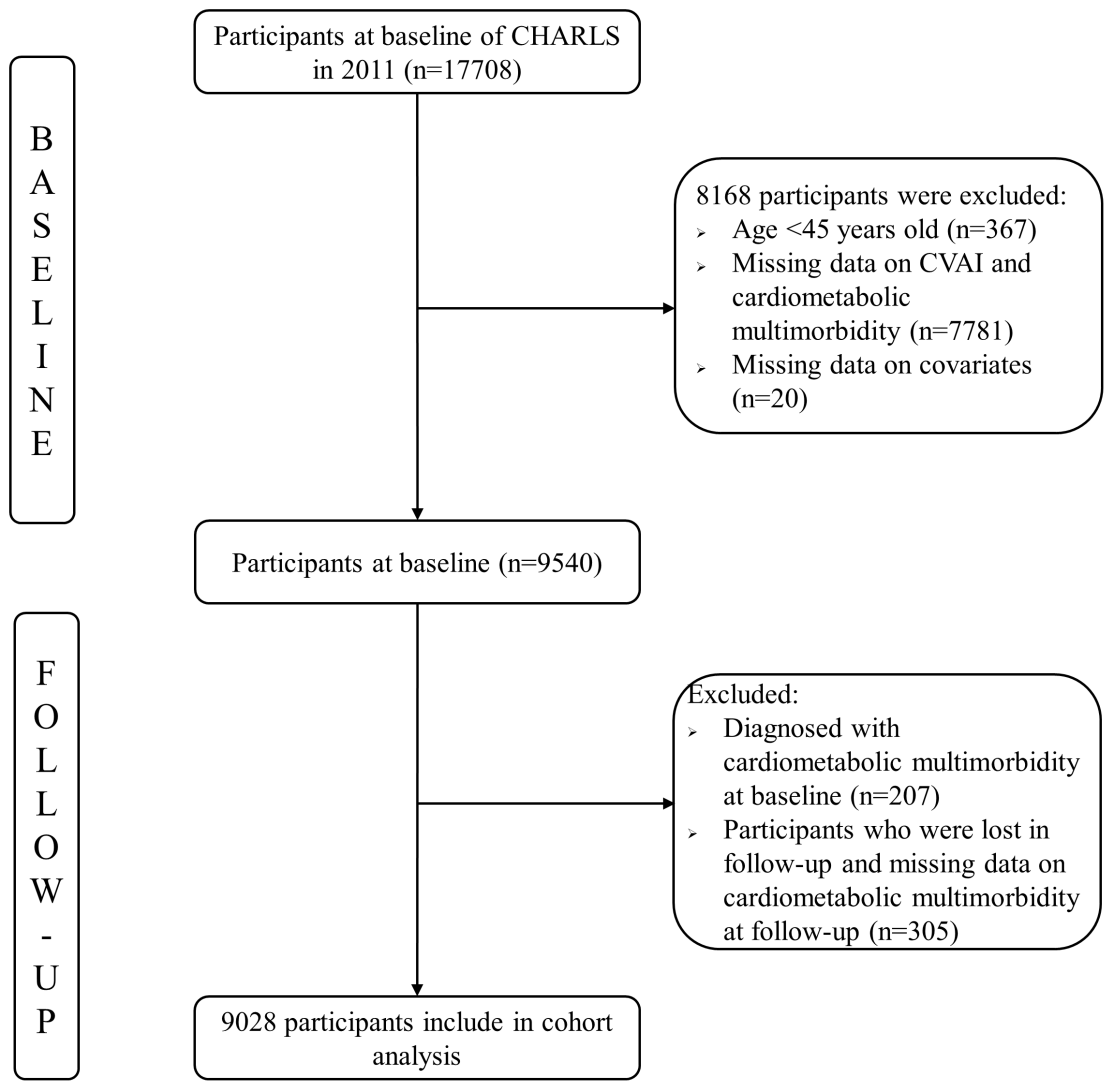
The selection process of the participants.

The data used in this study were obtained from a national cohort, the China Health and Retirement Longitudinal Study (CHARLS, 2011-2018 wave). The CHARLS is a nationally representative survey of middle-aged and older adults aged 45 years and older in China. Specific sampling and design details have been reported in the literature (25). We used 17708 participants from the 2011 CHARLS as potential study participants; a total of 9540 participants were included at baseline after excluding participants aged <45 years and those with missing data on CVAI, cardiometabolic multimorbidity, and covariates. We selected the 2013, 2015, and 2018 CHARLS as a follow-up survey, and 9028 participants were included in the cohort analysis after excluding participants with cardiometabolic multimorbidity at baseline, those lost to follow-up, and those with missing cardiometabolic multimorbidity data. CHARLS was approved by the Biomedical Ethics Review Committee of Peking University. **Figure 1** shows the selection of participants from the CHARLS cohort.

### 2.2 Measures

#### 2.2.1 CVAI

CVAI was measured using previously validated biomarker estimation formulas (21).

Males: CVAI = −267.93 + 0.68 * age + 0.03 * BMI(kg/m2) + 4.00 * WC(cm) + 22.00 * Log10TG(mmol/L) − 16.32 * HDL-C(mmol/L);

Females: CVAI = −187.32 + 1.71 * age + 4.23 * BMI(kg/m2) + 1.12 * WC(cm) + 39.76 * Log10TG(mmol/L) − 11.66 * HDL-C(mmol/L).

#### 2.2.2 Cardiometabolic multimorbidity

In alignment with previous studies (26), we focused on three cardiometabolic diseases: diabetes, heart disease, and stroke, excluding hypertension. Participants were asked ‘Have you been diagnosed with diabetes by a doctor?’, ‘Have you been diagnosed with heart attack, coronary heart disease, angina, congestive heart failure, or other heart problems by a doctor?’ and ‘Have you been diagnosed with stroke by a doctor?’, respectively. If the participants’ answer were yes, they were considered as having the disease. The presence of two or more of these three diseases is considered as cardiometabolic multimorbidity.

#### 2.2.3 Covariates

Socio-demographic characteristics, lifestyle, health status, and blood biomarkers were included as potential covariates. Socio-demographic characteristics included age, sex, residence, marital status, educational level, and family income (truncated by the median into low and high). Lifestyle included smoking, drinking, exercise, and participation in social activities. Health status included pain, self-rated health, health insurance, hypertension, respiratory disease, kidney disease, digestive disease, mental illness, current use of antihypertensive drugs, current use of antidiabetic drugs, and current use of antidyslipidemia drugs. Blood biomarkers included low-density lipoprotein cholesterol (LDL-C, unit: mg/dL), total cholesterol (unit: mg/dL), and fasting blood glucose (unit: mg/dL).

### 2.3 Statistical methods

In this study, we used the mean ± standard deviation (SD) to describe the distribution status of continuous variables, such as BMI and frequency (composition ratio), to characterize the distribution status of categorical variables, such as sex. To clearly distinguish the association between CVAI values and cardiometabolic multimorbidity, we divided the CVAI into four equal parts according to quartiles: Q1, Q2, Q3, and Q4. We used the Cox proportional hazard regression model to explore the association between CVAI quartiles and cardiometabolic multimorbidity, adjusting for covariates. Hazard ratios (HRs) and 95% confidence intervals (CIs) were used to show the strength of the associations. To demonstrate this association, after controlling for different covariates, we constructed three Cox models. Model 1 is not adjusted for any of the variables. Model 2 was adjusted for socio-demographic characteristics. Model 3 was adjusted for lifestyles, health status, and blood biomarkers, which was initially based on Model 2. We conducted subgroup analysis to explore sex and age heterogeneity in the association between CVAI and cardiometabolic multimorbidity. Additionally, two sensitivity analyses were conducted to further assess the robustness of the association. We first excluded participants with hypertension, respiratory disease, kidney disease, digestive disease, mental illness and reran the Cox regression analyses since the CVAIs of these participants may be extremely high or low. Second, we included the CVAI as a continuous variable in the model to determine whether the association between the specific value of the CVAI and cardiometabolic multimorbidity remained statistically significant to exclude artificial loss of information due to the classifications. Finally, we also conducted the receiver operating characteristic curve (ROC) analysis and calculated area under ROC (AUC) to assess the predictive capabilities of the CVAI for cardiometabolic multimorbidity. We used the maximal Youden index to determine ideal cutoff values for CVAI prediction of cardiometabolic multimorbidity by overall, age and sex. All statistical analyses were conducted using STATA 16.0, and the test level for statistical significance was set at *P* < 0.05.

## 3 Results

### 3.1 Descriptive statistics

The mean age of the 9028 participants included in the final analysis was 59.3 years (SD: 9.3), with a range of 45–96 years. Of these, 4183 (46.3%) were male, and 4845 (53.7%) were female. The mean CVAI was 98.39 (SD: 50.30). The chi-square test showed statistically significant differences in the distribution of CVAI according to age, sex, residence, marital status, educational level, smoking, drinking, social activity participation, hypertension, kidney disease, digestive disease, cardiometabolic multimorbidity, body mass index, waist circumference, triglyceride level, and high-density lipoprotein cholesterol level. The participants’ characteristics are shown in **Table 1**.

**Table 1:** Characteristics of participants according to CVAI.

Characteristics	Total	Quartiles of CVAI				*P* value
		Q1	Q2	Q3	Q4	
Total sample	9028 (100.0)	2257 (25.0)	2257 (25.0)	2257 (25.0)	2257 (25.0)	
Age, yeas						<0.001
45-59	4921 (54.5)	1421 (63.0)	1299 (57.6)	1194 (52.9)	1007 (44.6)	
≥60	4107 (45.5)	836 (37.0)	958 (42.4)	1063 (47.1)	1250 (55.4)	
Sex						<0.001
Male	4183 (46.3)	1320 (58.5)	1022 (45.3)	883 (39.1)	958 (42.4)	
Female	4845 (53.7)	937 (41.5)	1235 (54.7)	1374 (60.9)	1299 (57.6)	
Residence						<0.001
Rural	7545 (83.6)	2040 (90.4)	1948 (86.3)	1824 (80.8)	1733 (76.8)	
Urban	1483 (16.4)	217 (9.6)	309 (13.7)	433 (19.2)	524 (23.2)	
Marital status						0.003
Married	7922 (87.7)	2020 (89.5)	1989 (88.1)	1975 (87.5)	1938 (85.9)	
Unmarried	1106 (12.3)	237 (10.5)	268 (11.9)	282 (12.5)	319 (14.1)	
Educational level						<0.001
Illiteracy	2612 (28.9)	564 (25.0)	672 (29.8)	698 (30.9)	678 (30.0)	
Middle school below	3738 (41.4)	1007 (44.6)	961 (42.6)	872 (38.6)	898 (39.8)	
Middle school and above	2678 (29.7)	686 (30.4)	624 (27.6)	687 (30.4)	681 (30.2)	
Family income						0.228
Low	4316 (47.8)	1120 (49.6)	1072 (47.5)	1070 (47.4)	1054 (46.7)	
High	4712 (52.2)	1137 (50.4)	1185 (52.5)	1187 (52.6)	1203 (53.3)	
Smoking						<0.001
No	5509 (61.0)	1137 (50.4)	1397 (61.9)	1517 (67.2)	1458 (64.6)	
Yes	3519 (39.0)	1120 (49.6)	860 (38.1)	740 (32.8)	799 (35.4)	
Drinking						<0.001
No	6050 (67.0)	1315 (58.3)	1517 (67.2)	1618 (71.7)	1600 (70.9)	
Yes	2978 (33.0)	942 (41.7)	740 (32.8)	639 (28.3)	657 (29.1)	
Exercise						0.161
Hardly	5303 (58.7)	1343 (59.5)	1343 (59.5)	1280 (56.7)	1337 (59.2)	
Regularly	3725 (41.3)	914 (40.5)	914 (40.5)	977 (43.3)	920 (40.8)	
Social activity participation						<0.001
No	4441 (49.2)	1208 (53.5)	1185 (52.5)	1051 (46.6)	997 (44.2)	
Yes	4587 (50.8)	1049 (46.5)	1072 (47.5)	1206 (53.4)	1260 (55.8)	
Pain						0.678
No	5889 (65.2)	1464 (64.9)	1461 (64.7)	1468 (65.0)	1496 (66.3)	
Yes	3139 (34.8)	793 (35.1)	796 (35.3)	789 (35.0)	761 (33.7)	
Self-rated health						0.077
Excellent	603 (6.7)	128 (5.7)	146 (6.5)	165 (7.3)	164 (7.3)	
Very good	1393 (15.4)	344 (15.2)	336 (14.9)	342 (15.2)	371 (16.4)	
Good	4394 (48.7)	1158 (51.3)	1119 (49.6)	1069 (47.4)	1048 (46.4)	
Fair	2424 (26.8)	569 (25.2)	609 (27.0)	628 (27.8)	618 (27.4)	
Poor	214 (2.4)	58 (2.6)	47 (2.1)	53 (2.3)	56 (2.5)	
Health insurance						0.338
No	508 (5.6)	112 (5.0)	140 (6.2)	130 (5.8)	126 (5.6)	
Yes	8520 (94.4)	2145 (95.0)	2117 (93.8)	2127 (94.2)	2131 (94.4)	
Hypertension						<0.001
No	6884 (76.3)	1997 (88.5)	1882 (83.4)	1704 (75.5)	1301 (57.6)	
Yes	2144 (23.7)	260 (11.5)	375 (16.6)	553 (24.5)	956 (42.4)	
Respiratory disease						0.128
No	7970 (88.3)	1966 (87.1)	1987 (88.0)	2015 (89.3)	2002 (88.7)	
Yes	1058 (11.7)	291 (12.9)	270 (12.0)	242 (10.7)	255 (11.3)	
Kidney disease						0.001
No	8447 (93.6)	2073 (91.8)	2117 (93.8)	2123 (94.1)	2134 (94.6)	
Yes	581 (6.4)	184 (8.2)	140 (6.2)	134 (5.9)	123 (5.4)	
Digestive disease						<0.001
No	6659 (73.8)	1574 (69.7)	1663 (73.7)	1695 (75.1)	1727 (76.5)	
Yes	2369 (26.2)	683 (30.3)	594 (26.3)	562 (24.9)	530 (23.5)	
Mental illness						0.475
No	8908 (98.7)	2227 (98.7)	2224 (98.5)	2223 (98.5)	2234 (99.0)	
Yes	120 (1.3)	30 (1.3)	33 (1.5)	34 (1.5)	23 (1.0)	
Diabetes						<0.001
No	8167 (90.5)	2162 (95.8)	2120 (93.9)	2021 (89.5)	1864 (82.6)	
Yes	861 (9.5)	95 (4.2)	137 (6.1)	236 (10.5)	393 (17.4)	
Heart diseases						<0.001
No	7463 (82.7)	1966 (87.1)	1897 (84.1)	1849 (81.9)	1751 (77.6)	
Yes	1565 (17.3)	291 (12.9)	360 (15.9)	408 (18.1)	506 (22.4)	
Stroke						<0.001
No	8388 (92.9)	2166 (96.0)	2131 (94.4)	2082 (92.3)	2009 (89.0)	
Yes	640 (7.1)	91 (4.0)	126 (5.6)	175 (7.7)	248 (11.0)	
Cardiometabolic multimorbidity						<0.001
No	8828 (97.8)	2238 (99.2)	2228 (98.7)	2205 (97.7)	2157 (95.6)	
Yes	200 (2.2)	19 (0.8)	29 (1.3)	52 (2.3)	100 (4.4)	
Current use of antihypertensive drugs						<0.001
No	7346 (81.4)	2089 (92.6)	1979 (87.7)	1821 (80.7)	1457 (64.6)	
Yes	1682 (18.6)	168 (7.4)	278 (12.3)	436 (19.3)	800 (35.4)	
Current use of antidiabetic drugs						<0.001
No	8797 (97.4)	2237 (99.1)	2218 (98.3)	2208 (97.8)	2134 (94.6)	
Yes	231 (2.6)	20 (0.9)	39 (1.7)	49 (2.2)	123 (5.4)	
Current use of antidyslipidemia drugs						<0.001
No	8613 (95.4)	2224 (98.5)	2196 (97.3)	2156 (95.5)	2037 (90.3)	
Yes	415 (4.6)	33 (1.5)	61 (2.7)	101 (4.5)	220 (9.7)	
Body Mass Index, kg/m^2^	23.47 ± 3.68	20.47 ± 2.44	22.23 ± 2.47	24.12 ± 2.69	27.07 ± 3.31	<0.001
Waist circumference, cm	84.26 ± 12.52	72.11 ± 12.64	81.11 ± 7.47	87.45 ± 6.3	96.37 ± 7.55	<0.001
Triglyceride, mg/dl	133.09 ± 108.47	78.29 ± 36.4	102.29 ± 47.37	134.48 ± 64.05	217.3 ± 168.4	<0.001
HDL-C, mg/dl	51.33 ± 15.32	61.75 ± 16.08	54.5 ± 13.16	48.43 ± 12.11	40.62 ± 10.87	<0.001
LDL-C, mg/dl	116.64 ± 35.02	109.65 ± 30.88	117.04 ± 32.77	121.99 ± 33.83	117.89 ± 40.71	<0.001
Total cholesterol, mg/dl	193.87 ± 38.90	184.86 ± 35.20	190.35 ± 37.28	196.40 ± 37.56	203.88 ± 42.61	<0.001
Fasting blood glucose, mg/dl	109.62 ± 35.24	101.93 ± 23.50	106.50 ± 35.18	109.04 ± 31.84	121.01 ± 44.35	<0.001
CVAI	98.39 ± 50.30	37.85 ± 35.89	81.57 ± 8.71	113.17 ± 9.94	160.96 ± 24.01	<0.001

HDL-C, High-density lipoprotein cholesterol; LDL-C, Low-density lipoprotein cholesterol; CVAI, Chinese visceral adiposity index.

### 3.2 Cox hazard model

**Table 2** displays the associations between CVAI quartiles and cardiometabolic multimorbidity. In the unadjusted model, compared with the Q1 of CVAI, the Q3 and Q4 of CVAI were all significantly associated with a high incidence of cardiometabolic multimorbidity. After adjusting for age, sex, residence, marital status, educational level, and family income, the association between CVAI quartiles and cardiometabolic multimorbidity remained significant. In the fully-adjusted model, compared with participants in the Q1 of CVAI, the HRs (95% CIs) of participants in Q2, Q3, and Q4 of the CVAI for the risk of cardiometabolic multimorbidity were 1.358 (0.758 – 2.433), 2.203 (1.286 – 3.774), and 3.547 (2.100 – 5.992), respectively. Additionally, a linear association was observed between the CVAI quartiles and the incidence of cardiometabolic multimorbidity in all Cox models (*P <*0.001).

**Table 2:** Associations between CVAI and cardiometabolic multimorbidity.

Quartiles of CVAI	Model 1		Model 2		Model 3	
	HR (95%CI)	*P*-value	HR (95%CI)	*P*-value	HR (95%CI)	*P*-value
Q1 (reference)						
Q2	1.529 (0.857-2.727)	0.150	1.450 (0.812-2.592)	0.209	1.358 (0.758-2.433)	0.303
Q3	2.742 (1.622-4.638)	<0.001	2.515 (1.479-4.277)	0.001	2.203 (1.286-3.774)	0.004
Q4	5.308 (3.250-8.669)	<0.001	4.718 (2.861-7.780)	<0.001	3.547 (2.100-5.992)	<0.001
*P* for trend	<0.001		<0.001		<0.001	

Model 1 is not adjusted for any of the variables. Model 2 was adjusted for socio-demographic characteristics. Model 3 was adjusted for lifestyles, health status, and blood biomarkers, which was initially based on Model 2.

### 3.3 Subgroup analyses

The results of the subgroup analyses for sex and age are presented in **Table 3**. The association found between the CVAI quartiles and cardiometabolic multimorbidity remained statistically significant (*P*<0.05) in males and middle-aged adults aged 45-59 years. Meanwhile, only the Q4 of CVAI was associated with an increased risk of cardiometabolic multimorbidity in females and older adults aged 60 years or more. Additionally, there was no evidence of an interaction between the CVAI quartiles and sex or age in association with cardiometabolic multimorbidity (*P* for interaction >0.05).

**Table 3:** Subgroup analyses of CVAI and cardiometabolic multimorbidity.

Subgroup	HR (95%CI)	*P*-value	*P* for interaction
Male			
Q1 of CVAI (reference)			
Q2 of CVAI	1.727 (0.692-4.309)	0.242	
Q3 of CVAI	3.655 (1.600-8.347)	0.002	
Q4 of CVAI	5.541 (2.487-12.347)	<0.001	
Female			
Q1 of CVAI (reference)			
Q2 of CVAI	1.053 (0.494-2.244)	0.894	0.495
Q3 of CVAI	1.365 (0.671-2.775)	0.391	0.103
Q4 of CVAI	2.288 (1.130-4.636)	0.022	0.172
Age <60 years			
Q1 of CVAI (reference)			
Q2 of CVAI	0.896 (0.377-2.128)	0.803	
Q3 of CVAI	2.663 (1.307-5.428)	0.007	
Q4 of CVAI	3.223 (1.561-6.658)	0.002	
Age ≥60 years			
Q1 of CVAI (reference)			
Q2 of CVAI	1.725 (0.750-3.966)	0.200	0.235
Q3 of CVAI	1.609 (0.700-3.696)	0.263	0.462
Q4 of CVAI	3.387 (1.548-7.414)	0.002	0.979

### 3.4 Sensitivity analysis

In the first sensitivity analysis, we excluded participants with hypertension, respiratory disease, kidney disease, digestive disease, and mental illness, and performed a fully-adjusted Cox model. The results showed no substantial change in the association between CVAI quartiles and cardiometabolic multimorbidity (compared with Q1, the HRs of the participants in Q3 and Q4 were 2.708 [95%CI= 1.039-7.055], 3.966 [95%CI= 1.505-10.450]). Additionally, we conducted a second sensitivity analysis using CVAI as a continuous variable. The results suggested that the elevated CVAI values were associated with an increased risk of cardiometabolic multimorbidity (HR= 1.011, 95%CI= 1.007-1.014). The results of both sensitivity analyses suggested that the association between CVAI and cardiometabolic multimorbidity was robust.

### 3.5 Predictive capability of the CVAI for cardiometabolic multimorbidity

The results of predictive capability of the CVAI for cardiometabolic multimorbidity are presented in **Table 4**. The AUC of overall, male group, female group, the aged <60 years, and the aged ≥60 was 0.685 (95% CI = 0.649-0.722), 0.702 (95% CI = 0.649-0.759), 0.668 (95% CI = 0.618-0.728), 0.701 (95% CI = 0.647-0.756), and 0.660 (95% CI = 0.610-0.711), respectively. The ideal cutoff value for CVAI prediction of cardiometabolic multimorbidity was 121.388 in total sample. For each group, the following cutoff values were 102.378 in male, 136.554 in female, 101.927 in the aged <60 years group, and 132.568 in the aged ≥60 years group. More details are displayed in **Table 4**.

**Table 4:** Predictive capability of the CVAI for cardiometabolic multimorbidity.

Sample	AUC	95%CI of AUC	Sensitivity	Specificity	Cut-off value	*P*-value
Overtall	0.685	0.649-0.722	0.600	0.691	121.388	<0.001
By sex						
Male	0.702	0.649-0.759	0.741	0.604	102.378	<0.001
Female	0.668	0.618-0.728	0.487	0.772	136.554	<0.001
By age						
Age <60 years	0.701	0.647-0.756	0.742	0.600	101.927	<0.001
Age ≥60 years	0.660	0.610-0.711	0.559	0.711	132.568	<0.001
By sex and age						
Male aged <60 years	0.753	0.675-0.831	0.872	0.583	98.679	<0.001
Male aged ≥60 years	0.653	0.574-0.732	0.833	0.416	78.803	<0.001
Female aged <60 years	0.654	0.580-0.728	0.800	0.479	89.305	<0.001
Female aged ≥60 years	0.652	0.584-0.720	0.609	0.671	136.535	<0.001

## 4 Discussion

Our study explored the association between CVAI and cardiometabolic multimorbidity among middle-aged and older Chinese adults based on a national cohort. We found a linear association between the CVAI quartiles and the risk of cardiometabolic multimorbidity. Compared with the participants in Q1 of the CVAI, those in Q3 and Q4 had a 135% and 278% higher risk of future cardiometabolic multimorbidity, respectively. The results of ROC analyses indicate that CVAI has good predictive capability for cardiometabolic multimorbidity. This study suggests that the CVAI may be a reliable biomarker for predicting the occurrence of cardiometabolic multimorbidities in clinical settings.

The association between CVAI and cardiometabolic diseases has been confirmed in several previous studies. For example, a recent study based on the CHARLS found that an increase in the CVAI predicted the risk of developing future strokes among middle-aged and older adults (23). A U-shaped relationship was also found between CVAI and type 2 diabetes mellitus (27). Additionally, evidence suggests that the CVAI is associated with cardiometabolic multimorbidity. In a cohort study involving more than 2,000 patients with type 2 diabetes, elevated CVAI was found to increase the risk of cardiovascular events (28). Additionally, the risk of stroke in patients with metabolic syndrome increased with increased CVAI (29). A cross-sectional study of Chinese older adults showed a linear association between CVAI and the prevalence of hypertension and diabetes mellitus (30). This observational study provides strong support for our findings. According to the capacity–load model, the risk of cardiometabolic diseases is a combination of the interaction between the metabolic capacity and the load of the body (2). Obesity is thought to increase the metabolic load, leading to impaired metabolic capacity of the body and imbalances in metabolic homeostasis, leading to an increased risk of cardiometabolic diseases (31, 32). Visceral fat is mainly located in the abdominal cavity, and its increase can cause fat cells to spill over into the surrounding organs (liver, kidneys, and heart), resulting in insulin resistance, which impairs the metabolic function of these organs (20). Furthermore, elevated CVAI produces insulin resistance, leading to renal cytokine imbalance and damage to the glomerular basement membrane, initiating metabolic dysfunction in the kidneys (33–35). Additionally, a UK Biobank-based study found that pericardial adipose tissue objectively measured using cardiovascular magnetic resonance imaging significantly and positively correlated with abnormal cardiovascular structure and function (36). It has also been found that increased abdominal adipose tissue induces the production of inflammatory cytokines, leading to an inflammatory response and oxidative stress that impairs vascular endothelial function (37).

The results of the subgroup analysis revealed interesting findings. First, this study found that the association between the CVAI quartiles and cardiometabolic multimorbidity was stronger in males than in females, wherein both the Q3 and Q4 of the CVAI were associated with an increased risk of cardiometabolic multimorbidity, whereas in females only Q4 showed an association. A previous animal study based on obese mice found a stronger pro-inflammatory state in the adipose endothelial cells of male mice than in female mice, indicating that male mice are put at higher risk due to obesity (38). Additionally, animal experiments have shown that obese female mice have a higher glucose tolerance than male mice due to the anti-inflammatory properties of estrogen, which reduces immune cell infiltration and oxidative stress in the adipose tissue of female mice (39). Evidence from animal studies may partially explain our findings, but more population-based studies are needed to further elucidate the causes of these differences due to sex. Another interesting finding was that the association between CVAI quartiles and cardiometabolic multimorbidity was stronger in the middle-aged group aged 45-59 years, compared to that of older adults aged ≥ 60 years. Two previous studies reported similar findings in that the CVAI was more strongly associated with new-onset diabetes (27) and stroke (40) in the middle-aged adults compared to older adults. There exists the persistence of the “obesity paradox” in older adults, wherein the all-cause mortality is lower in the overweight BMI group compared with the normal BMI group (41). It has also been found that overweight and mildly obese individuals have a lower prevalence of cardiovascular disease and a better prognosis (42, 43). This may partially support our finding that the Q4 of the CVAI is associated with a higher risk of cardiometabolic multimorbidity in the older adult group, but not Q3. Given that only a few studies have directly explored the associations and biological mechanisms of CVAI with health outcomes in older adults, future in-depth animal experiments or long-term cohort studies are required to validate our findings.

This study has strengths in terms of its research design and analytical strategy. First, it used population-representative data from a large national epidemiological survey, which may allow our findings to be extrapolated to the entire Chinese middle-aged and elderly population. The findings of this study have a high practical value in guiding the early warning and prevention of cardiometabolic morbidity in middle-aged and elderly people, such as the incorporation of CVAI as a biomarker for cardiometabolic morbidity in primary health care. Additionally, several confounders in our statistical analyses were controlled, and subgroup analyses for age and sex were also performed, which allowed us to detect possible differences in different groups and to estimate more accurately the association between CVAI and cardiometabolic multimorbidity. A sensitivity analysis was also conducted to test the robustness of the findings. Finally, we also analyzed the predictive capability and optimal cutoff value of CVAI for cardiometabolic multimorbidity, which can be used by healthcare professionals. However, this study has some limitations. First, although we used a cohort study with a high level of evidence to explore the association between CVAI and cardiometabolic multimorbidity, observational studies were limited since these could not infer causality. In the future, causal inference methods, such as Mendelian randomization or experimental study designs, should be used to elucidate the causal relationship between the two. Second, due to database limitations, the CHARLS did not have records on the dietary, precise physical activity, specific drug usage (such as statins and antiplatelet drugs) from the participants at the time of the survey, which may lead to the omission of covariates in our study, resulting in confounding bias. Third, we excluded participants with missing variables in the baseline survey and those lost to follow-up, which created a selection bias. Finally, cardiometabolic multimorbidity was self-reported. Since the participants were tasked to report their physician-diagnosed disease, this may have been subject to recall bias.

## 5 Conclusions

Elevated CVAI was significantly associated with a higher risk of cardiometabolic multimorbidity in middle-aged and older Chinese adults. The CVAI was found to be a valid biomarker with good predictive capability for cardiometabolic multimorbidity and can be used by primary healthcare organizations in the future for early warning, prevention, and intervention in cardiometabolic multimorbidity.

## Data availability statement

The datasets presented in this article are not readily available because the data of this study can be obtained on the official website of CHARLS. Requests to access the datasets should be directed to http://charls.pku.edu.cn/.

## Ethics statement

The studies involving humans were approved by Biomedical Ethics Review Committee of Peking University. The studies were conducted in accordance with the local legislation and institutional requirements. The participants provided their written informed consent to participate in this study.

## Author contributions

XY: Conceptualization, Formal Analysis, Methodology, Software, Writing – original draft. GZ: Formal Analysis, Methodology, Writing – original draft. CH: Validation, Writing – review & editing. PW: Validation, Writing – review & editing. JL: Supervision, Writing – review & editing. MZ: Supervision, Writing – review & editing, Funding acquisition, Project administration.
